# Histone Deacetylase Expressions in Hepatocellular Carcinoma and Functional Effects of Histone Deacetylase Inhibitors on Liver Cancer Cells In Vitro

**DOI:** 10.3390/cancers11101587

**Published:** 2019-10-18

**Authors:** Kim Freese, Tatjana Seitz, Peter Dietrich, Serene M.L. Lee, Wolfgang E. Thasler, Anja Bosserhoff, Claus Hellerbrand

**Affiliations:** 1 Institute of Biochemistry (Emil-Fischer Zentrum), Friedrich-Alexander University Erlangen-Nürnberg, Fahrstr. 17, D-91054 Erlangen, Germany; kim.freese@fau.de (K.F.); tatjana.seitz@fau.de (T.S.); peter.dietrich@fau.de (P.D.); anja.bosserhoff@fau.de (A.B.); 2 Medical Clinic 1, Department of Medicine, University Hospital Erlangen, Friedrich-Alexander-University, D-91054 Erlangen, Germany; 3 Biobank under the administration of the Human Tissue and Cell Research Foundation. Department of General Visceral- and Transplantation Surgery, Ludwig-Maximilians-University Munich, D-81377 Munich, Germany; serene.lee@med.uni-muenchen.de; 4 Hepacult GmbH, Am Klopferspitz 19, D-82152 Planegg/Martinsried, Germany; wolfgang.thasler@swmbrk.de; 5 Comprehensive Cancer Center (CCC) Erlangen-EMN, D-91054 Erlangen, Germany

**Keywords:** histone deacetylase, hepatocellular carcinoma, sorafenib, histone deacetylase inhibitor

## Abstract

Hepatocellular carcinoma (HCC) is a leading cause for deaths worldwide. Histone deacetylase (HDAC) inhibition (HDACi) is emerging as a promising therapeutic strategy. However, most pharmacological HDACi unselectively block different HDAC classes and their molecular mechanisms of action are only incompletely understood. The aim of this study was to systematically analyze expressions of different HDAC classes in HCC cells and tissues and to functionally analyze the effect of the HDACi suberanilohydroxamic acid (SAHA) and trichostatin A (TSA) on the tumorigenicity of HCC cells. The gene expression of all HDAC classes was significantly increased in human HCC cell lines (Hep3B, HepG2, PLC, HuH7) compared to primary human hepatocytes (PHH). The analysis of HCC patient data showed the increased expression of several HDACs in HCC tissues compared to non-tumorous liver. However, there was no unified picture of regulation in three different HCC patient datasets and we observed a strong variation in the gene expression of different HDACs in tumorous as well as non-tumorous liver. Still, there was a strong correlation in the expression of HDAC class IIa (HDAC4, 5, 7, 9) as well as HDAC2 and 8 (class I) and HDAC10 (class IIb) and HDAC11 (class IV) in HCC tissues of individual patients. This might indicate a common mechanism of the regulation of these HDACs in HCC. The Cancer Genome Atlas (TCGA) dataset analysis revealed that HDAC4, HDAC7 and HDAC9 as well as HDAC class I members HDAC1 and HDAC2 is significantly correlated with patient survival. Furthermore, we observed that SAHA and TSA reduced the proliferation, clonogenicity and migratory potential of HCC cells. SAHA but not TSA induced features of senescence in HCC cells. Additionally, HDACi enhanced the efficacy of sorafenib in killing sorafenib-susceptible cells. Moreover, HDACi reestablished sorafenib sensitivity in resistant HCC cells. In summary, HDACs are significantly but differently increased in HCC, which may be exploited to develop more targeted therapeutic approaches. HDACi affect different facets of the tumorigenicity of HCC cells and appears to be a promising therapeutic approach alone or in combination with sorafenib.

## 1. Introduction

Hepatocellular carcinoma (HCC) is the fourth leading cause of cancer-related death worldwide and has a rising incidence [1]. Despite the burden HCC causes, knowledge on the molecular mechanisms of the development and progression of this disease is still limited and treatment options are not optimal. In most cases, HCC is diagnosed in already advanced stages with limited therapeutic options [2]. Sorafenib is the first-line treatment for advanced-stage HCC patients [3]. Although treatment with this multi-target tyrosine kinase inhibitor is associated with overall survival benefits in this group of HCC patients, the response rate is not satisfactory. Therefore, new therapeutic targets as well as an understanding of the molecular mechanisms associated with sorafenib resistance are highly needed to improve treatment options for HCC patients.

It is increasingly recognized that cancer development and progression is significantly affected by epigenetic mechanisms. Among these, histone deacetylases (HDACs) have been shown to play a key role in different hallmarks of cancer including resistance to apoptosis and chemotherapy resistance [4,5]. 

Today, 11 human zinc-dependent HDACs are known (HDAC1-11). Based on their general sequence homology to yeast, they can be clustered into different classes: class I: HDAC1, 2, 3, 8; class IIa: HDAC4, 5, 7, 9; class IIb: HDAC6, 10; and class IV: HDAC11. Class III comprises non-zinc-dependent HDACs, so called sirtuines, which we did not aim to analyses in this study. 

Previous studies have shown the overexpression of individual HDACs in HCC (subtypes) and their impact on HCC progression. For example, Ler et a. found that HDAC1 and HDAC2 were upregulated in the majority of HCC tissues, and that this upregulation was associated with cancer-specific mortality [6]. Quint et al. described an increased expression of HDACs 1–3 in HCC and a high concordance of expression levels with each other, but only HDAC2 expression had an impact on patient survival [7]. Furthermore, the targeted inhibition of defined HDACs such as HDAC4, HDAC5 or HDAC6 has been shown to inhibit HCC growth and metastasis [8,9,10]. Therefore, the application of HDAC inhibitors (HDACi) is an emerging approach with promising results in preclinical settings [11]. 

Suberoylanilide hydroxamic acid (SAHA) is an irreversible pan HDACi, which was approved for the treatment of cutaneous T-cell lymphoma [12]. SAHA effectively inhibits class I and II HDACs with higher IC50 for HDAC 4, 7 and 9 [13]. Trichostatin A (TSA) is a reversible pan HDACi with higher affinity and thus inhibition of class I, HDAC5 and class IIb HDACs and less effectiveness against HDAC 4, 7, 9 and 11 [14,15]. In HCC, SAHA and TSA have been shown to induce different cell death molecular cascades [11,16,17,18,19]. However, studies on further anti-tumorigenic effects as well as effects on chemotherapy resistance are very sparse.

The aim of this study was to systemically analyze the expression of all 11 classical (zinc-dependent) HDACs in HCC cells and tissues. Furthermore, we evaluated the effects of TSA and SAHA on different facets of the tumorigenicity of human HCC cells in functional assays, while also examining the combined effects of HDACi with sorafenib in wildtype as well as sorafenib-resistant HCC cells.

## 2. Results

### 2.1. HDAC Expression in HCC Cells and Tissues

**Figure 1:**
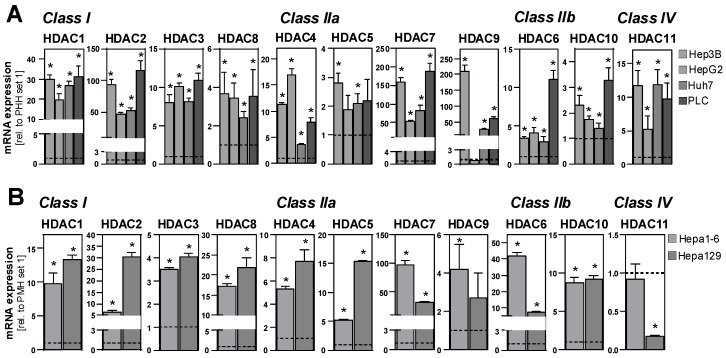
Histone deacetylase (HDAC) expression in hepatocellular carcinoma (HCC) cells compared with hepatocytes. (**A**) HDAC mRNA expression levels in four human HCC cell lines (Hep3B, HepG2, Huh7 and PLC) relative to expression in primary human hepatocytes (PHHs; set at a value of 1). (**B**) HDAC mRNA expression in two murine HCC cell lines (Hepa1-6 and Hepa129) relative to expression in primary murine hepatocytes (PMH; set at a value of 1). (*: *p* < 0.05 compared to PHH or PMH.).

First, we analyzed the mRNA expression of all HDAC class I (HDAC 1, 2, 3 and 8), class IIa (HDAC 4, 5, 7 and 9), class IIb (HDAC 6 and 10) and class IV (HDAC 11) in four different human HCC cell lines (Hep3B, HepG2, Huh7 and PLC) and primary human hepatocytes (PHH) with quantitative PCR. The expression levels of all 11 HDACs were significantly higher in all HCC cell lines compared to PHH (Figure 1A). Only in HepG2 cells, HDAC9 was not significantly increased compared with PHH (Figure 1A). Similarly, the expression of HDAC classes I and II was significantly increased in the 2 murine HCC cell lines Hepa1-6 and Hepa129 compared with primary murine hepatocytes (Figure 1B). Only HDAC11 levels were not increased or even lower, respectively, in murine HCC cells compared with hepatocytes (Figure 1B).

**Figure 2:**
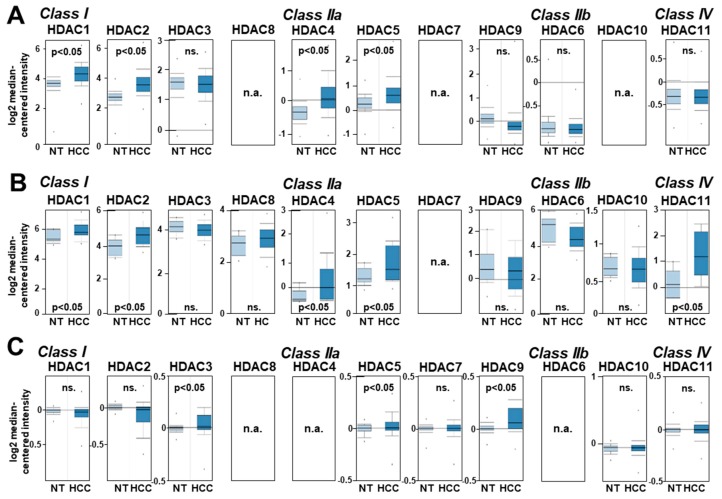
HDAC expression in human HCC compared with non-tumorous liver tissues. (**A–C**) HDAC mRNA levels in non-tumorous liver tissues (NTs) as compared to HCC tissues from patients. Data were obtained from the Oncomine^TM^ cancer microarray database using the datasets ‘Roessler Liver2’ (n = 445) (C), ‘Wurmbach Liver’ (n = 75) (D), and ‘Guichard Liver’ (n = 185) (E). (*: *p* < 0.05 compared to non-tumorous tissue; n.a.: no data available.)

The expression levels of different HDACs in HCC patients were analyzed using the Oncomine^TM^ human cancer microarray database [20]. In one dataset comprising 445 HCC patients (Roessler Liver 2 [21]), HDAC 1, 2 (class I) and HDAC 4, 5 and 9 (class IIa) but not HDAC 3, 6 and 11 were found to be significantly upregulated in HCC as compared to non-HCC liver tissues (Figure 2A). (HDAC 8, 7 and 10 expression data were not available in this dataset.) In a second dataset comprising 75 HCC patients (Wurmbach Liver [22]), HDAC 1 and 2, HDAC 4 and 5 and HDAC11 were significantly upregulated, whereas HDAC 3, 8, 9, 6 and 10 were not altered compared to non-neoplastic liver tissue (Figure 2B). (No data were available for HDAC7 in the Wurmbach Liver dataset.) Next, a third dataset with 185 HCC patient mRNA expression data was analyzed for HDAC expression in HCC (Guichard Liver, [23]). Here, HDAC3 (class I) and HDAC5 and 7 (class IIa) were significantly upregulated in patient HCC tissues, whereas HDAC 1, 2, 7, 10 and 11 expression levels did not significantly differ from non-tumorous liver tissues (Figure 2C). (HDAC 8, 4 and 6 expression data were not available in this dataset.)

There was a strong variation in HDAC expression in the non-tumorous liver tissues. Previous studies revealed a pathological imbalance between the acetylation and deacetylation of histones in liver fibrosis [24]. Therefore, we used the University of California Santa Cruz (UCSC) Xena platform with a dataset of 50 non-tumorous liver tissue samples [25] to analyze the correlation between the RNA expression levels of the different HDACs and collagen I (alpha 1), the most abundant extracellular matrix protein in liver fibrosis [25,26]. This analysis revealed a significant correlation of the expression of HDAC 1 (class I), HDAC 4, 7 and 9 (class IIa), as well as HDAC 6 (class IIa) and HDAC 11 (class IV), with the expression of collagen I in non-tumorous liver tissue (Table 1). These data indicate that HDAC expression is increased in fibrotic liver tissue and, thus, could be an explanation for the high variation in HDAC levels in the non-tumorous liver tissues of HCC patients. Furthermore, this finding could explain why the different HDACs are not consistently upregulated in HCC compared to non-tumorous liver tissues in the different datasets of patients.

In addition to the non-tumorous liver tissues, there was also a high variation in the expression levels of all HDAC classes within the HCC tissues. We wanted to analyze whether the variation in the different HDACs occurs independent from each other or whether there is a correlation between the expression levels of the different HDACs in HCC tissues. Therefore, we applied RT-qPCR analysis to determine the mRNA expression levels of the different HDACs in eleven human HCC tissue samples. Similar to in the in silico analysis, the mRNA expression levels of all HDACs showed a high variation in the HCC tissues of the different patients (Figure S1). Interestingly, we found a significant correlation between the expression levels of all four HDAC class IIa members (HDAC 4, 5, 7 and 9) (Table 2). Moreover, there was a significant correlation of HDAC 2 (class I), HDAC 10 (class IIb) and HDAC 11 (class IV) with most other HDACs. In contrast, the expression levels of HDAC 1 and HDAC 3 (class I) or HDAC 6 (class IIb) did not correlate with the expression of other HDACs (Table 2).

**Figure 3:**
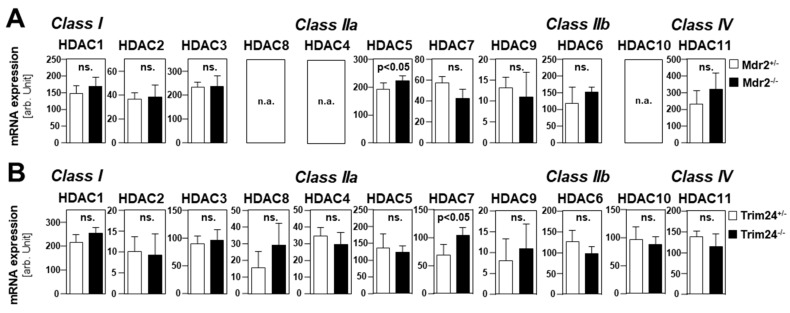
HDAC expression levels in genetically induced murine HCC models. (**A**,**B**) In silico analysis of mRNA expression levels for different HDACs was performed using GEO/GSE datasets (https://www.ncbi.nlm.nih.gov/geoprofiles). (**A**) RNA expression levels in pre-cancerous stages in the murine Mdr2 knockout HCC model in heterozygous ((Mdr^+/-^, n = 6) and homozygous (Mdr^-/-^, n = 6) knockouts (*p < 0.05 vs hetero). (**B**) RNA expression levels in livers from wild-type (ctrl, n = 5) as compared to HCC tumors (HCC, n = 5) derived from the Trim24-deficient spontaneous murine HCC model (ns: non-significant vs ctrl; *p < 0.05 vs ctrl).

Next, we used GEO/GSE datasets (https://www.ncbi.nlm.nih.gov/gds) to gain insights into the gene expression of HDACs during HCC development. Once, a precancerous dataset comparing heterozygous and homozygous Mdr2 knockout mice was analyzed. Previous studies had shown that the Mdr2-KO mouse is a valid model for human HCC development [27]. In this dataset, only HDAC 5 mRNA expression was slightly increased in the liver of homozygous as compared to heterozygous knockout mice (Figure 3A). The expression levels of the other HDACs did not show significant differences (Figure 3A). Moreover, HDAC expression levels were analyzed in a GEO/GSE dataset containing data on Trim24-deficient HCC samples and non-tumorous control liver tissues. Furthermore, Trim24-deficient mice spontaneously develop HCC [28]. In addition, in this model, the expression levels of most HDACs did not differ significantly between HCC and non-tumorous wild-type liver tissue (Figure 3B). Only HDAC 7 expression was slightly higher in tumorous as compared to non-tumorous mouse livers (Figure 3B). Together, these data indicated that the upregulation of HDACs does not occur during HCC development but rather in advanced liver cancer.

### 2.2. Correlation of HDAC Expression with Clinical Prognosis of HCC Patients

**Figure 4:**
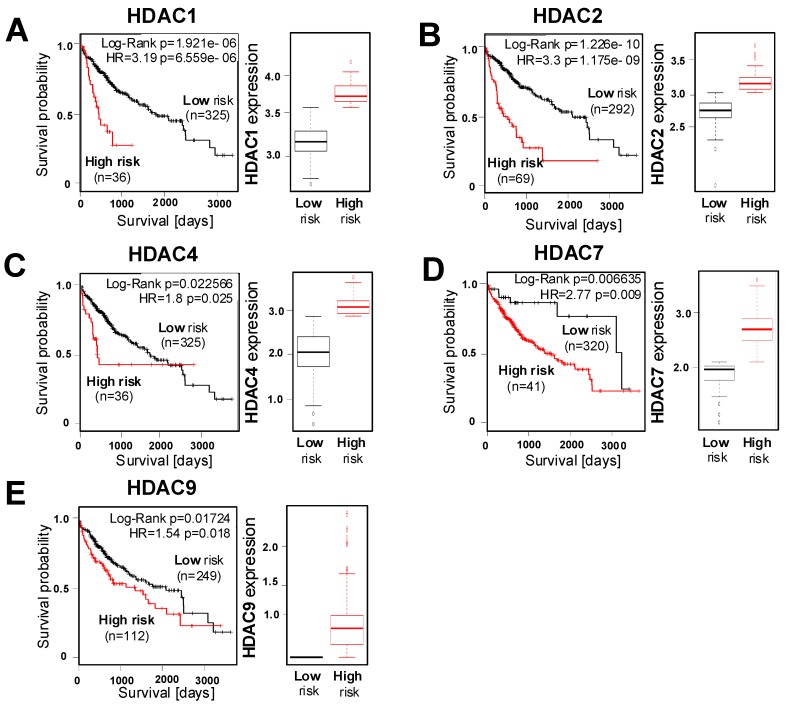
HDAC expression levels and prognosis of HCC patients. (**A–E**) The representative Kaplan–Meier survival curve analysis was performed using the SurvExpress Biomarker validation database for a TCGA HCC (LIHC) dataset (n = 361) for overall survival (left panel) with stratification into ‘low-risk’ and ‘high-risk’ groups based on the prognostic index for different HDACs and the quantification of the corresponding mRNA expression (right panel).

Next, we wanted to assess the correlation between tumorous HDAC expression and survival of HCC patients using the "SurvExpress" Biomarker validation for the cancer gene expression database [29]. Based on the prognostic index, also known as the risk score, patients were stratified into "low-risk" and "high-risk" groups [29]. Computational stratification revealed a significant overexpression of HDAC 1 and 2 (class I), as well as HDAC 4, 7 and HDAC 9 (class IIa), in the high- compared to low-risk groups in the LIHC-TCGA HCC dataset (n = 361) (Figure 4A–E). Furthermore, the analysis of this dataset revealed a reduced overall survival in HCC patients with high HDAC 1 and 2 or HDAC 4, 7 and 9 expression (Figure 4A–E).

Furthermore, in the TCGA Liver Cancer dataset (n = 422), computational stratification revealed the significant overexpression of HDAC 1 and 2 as well as HDAC 7 and HDAC 9 in the high-risk group and a correlation of this overexpression with reduced overall survival (Figure S2). 

Moreover, the analysis of the Hoshida Golub Liver GSE10143 dataset (n = 162) showed the higher expression of HDAC 1, HDAC 2, and HDAC 9 in the high-risk group, and that this overexpression correlated with poor survival (Figure S2). In summary, these results indicate that enhanced HDAC expression in HCC cells is associated with a poor prognosis.

### 2.3. Effect of HDAC Inhibition on the Viability of HCC Cell Lines and Primary Hepatocytes

**Figure 5:**
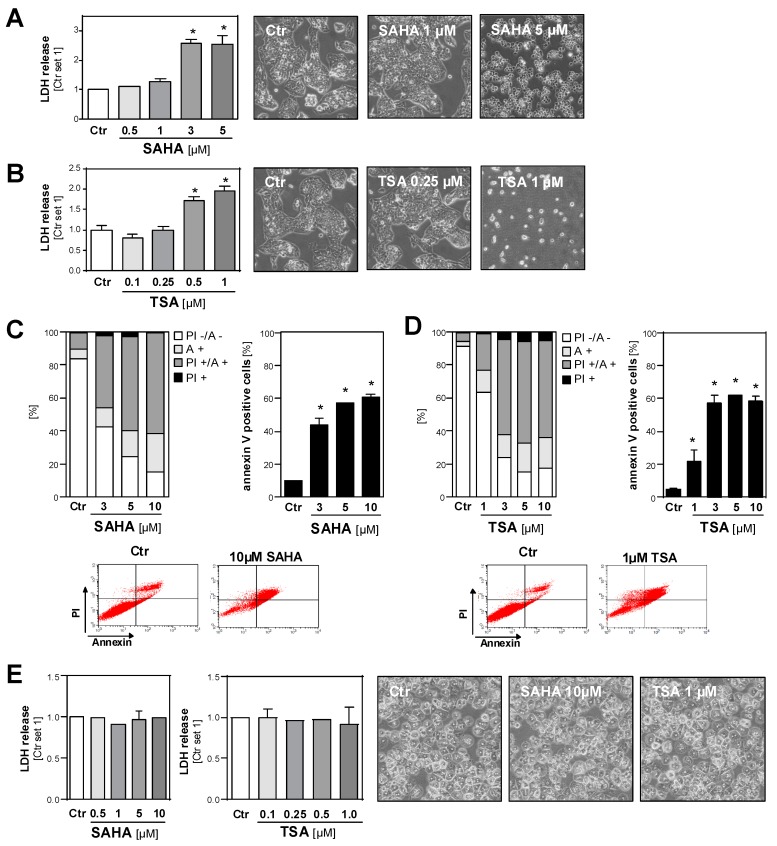
Effects of HDAC inhibition on the viability of HCC cells and primary hepatocytes. (**A**,**B**) Lactate dehydrogenase (LDH) release into the supernatant (left panel) and representative light microscopical images (right panels) of human HCC cells (HepG2) after 72 h treatment with different doses of SAHA (A) or TSA (B). Control cells (Ctr.) treated with solvent (DMSO) only were set as 1. (**C**,**D**) Propidium iodide/annexin (PI/A) FACS analysis of HepG2 cells after 24 h treatment with SAHA (C) or TSA (D). Left panel: proportions of viable (PI -/ A-), early (A +) and late apoptotic (PI +/ A+) and necrotic cells (PI +); right panel: quantification of annexin-positive late apoptotic cells; lower panel: representative FACS images. (**E**) LDH release into the supernatant (left panels) and light microscopical images (right panels) of primary human hepatocytes treated with different doses of SAHA or TSA for 72 h. Control cells (Ctr.) treated with solvent (DMSO) only were set at a value of 1. (*: *p* < 0.05 compared to Ctr.).

Next, we wanted to systematically analyze the effects of HDAC inhibition on the tumorigenicity of different HCC cell lines. To dissect the cytotoxic and functional effects, we first determined the dose range of toxicity of the HDAC inhibitors SAHA and TSA. The analysis of LDH release into the supernatant and microscopical analysis revealed that 72 h incubation with SAHA doses up to 1 µM and TSA doses up to 0.25 µM, respectively, did not induce toxic effects in HCC cells (Figure 5A,B and Figure S3A,B). Fluorescence-activated cell sorting (FACS) analysis (propidium iodide/annexin) showed a dose-dependent induction of necrosis and apoptosis in HCC cells after treatment with SAHA (Figure 5C) or TSA (Figure 5D) for 24 h. Notably, treatment with 4–10 fold higher doses of both HDACi for 72 h did not cause toxic effects in primary human hepatocytes (Figure 5E).

### 2.4. Functional Effects of HDAC Inhibition on HCC Cell Lines

**Figure 6:**
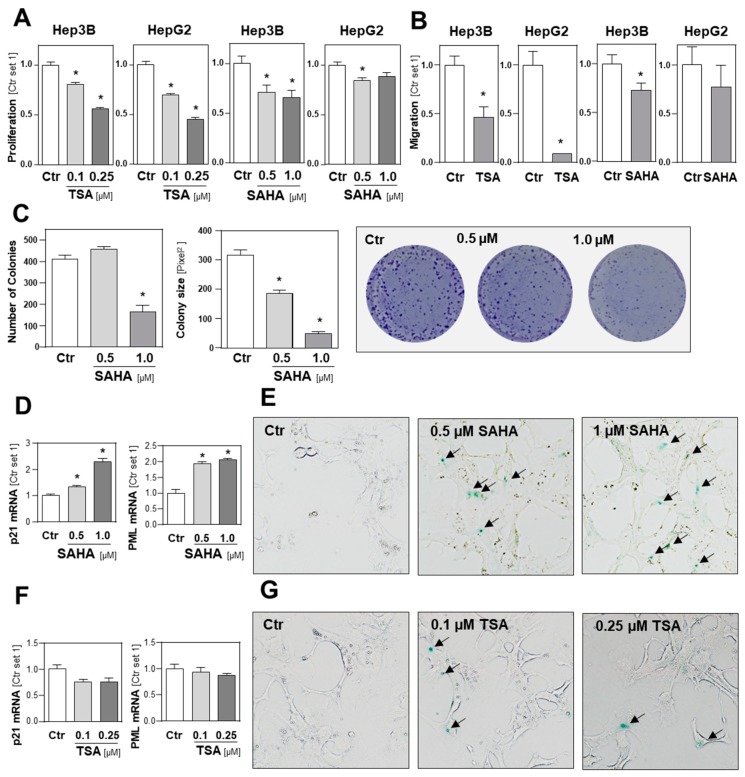
Functional effects of HDAC inhibition on HCC cells. (**A**) Proliferation of HCC cells (Hep3B, HepG2) after 32 h treatment with nontoxic doses of trichostatin A (TSA) or suberoylanilide hydroxamic acid (SAHA). Control cells (Ctr.) treated with solvent (DMSO) only were set at a value of 1. (**B**) Migratory activity of HCC cells after 4 h treatment with nontoxic doses of TSA (0.1 µM) or SAHA (1 µM). (**C**) Anchorage-dependent clonogenic assay with Hep3B cells after HDACi treatment. Quantification of colony numbers (left panel), colony size (middle panel) and representative images (right panel). (**D**) p21, mRNA expression, (**F**) PML mRNA expression and (**E**,**G**) ß-galactosidase (ß-Gal) staining of Hep3B cells after 72 h treatment with SAHA or TSA. Arrows indicate green stained ß-Gal-positive cells. (*: *p* < 0.05 compared to Ctr.).

Next, we analyzed the effect of HDACi on HCC cells in functional assays using subtoxic SAHA and TSA concentrations. A TSA dose of 0.1 µM significantly impaired the growth of HepG2 and Hep3B cells and, at a dose of 0.25 µM TSA, abrogated the proliferation of both HCC cells lines (Figure 6A). The maximal SAHA dose of 1 µM reduced the growth of Hep3B cells to approximately 60% of the control cells, while the same SAHA dose only slightly affected the growth of HepG2 cells (Figure 6A). Next, we analyzed the impact of HDACi on the migration of HCC cells in transwell Boyden chamber assays and observed that SAHA and TSA significantly reduced the directed migration of HCC cells (Figure 6B). Furthermore, SAHA and TSA treatment dose-dependently reduced the number and size of colonies formed by HCC cells in colony formation assays (Figure 6C; Figure S3C,D). 

Next, we analyzed the effect of HDACi on features of cellular senescence. We found that SAHA treatment caused a dose-dependent induction of p21 and promyelocytic leukemia protein (PML) expression in HCC cells (Figure 6D). Furthermore, we found a significant increase in ß-galactosidase (ß-Gal)-positive cells after SAHA treatment (Figure 6E). In contrast, TSA treatment did not change p21 and PML expression levels in HCC cells and did not significantly affect the number of ß-Gal-positive HCC cells (Figure 6F,G). In summary, these data also indicate that subtoxic HDACi concentrations exhibit a significant inhibitory effect on the growth and migratory activity of HCC cells.

### 2.5. Effects of HDAC Inhibition on HCC Cell Lines in Combination with Sorafenib

**Figure 7:**
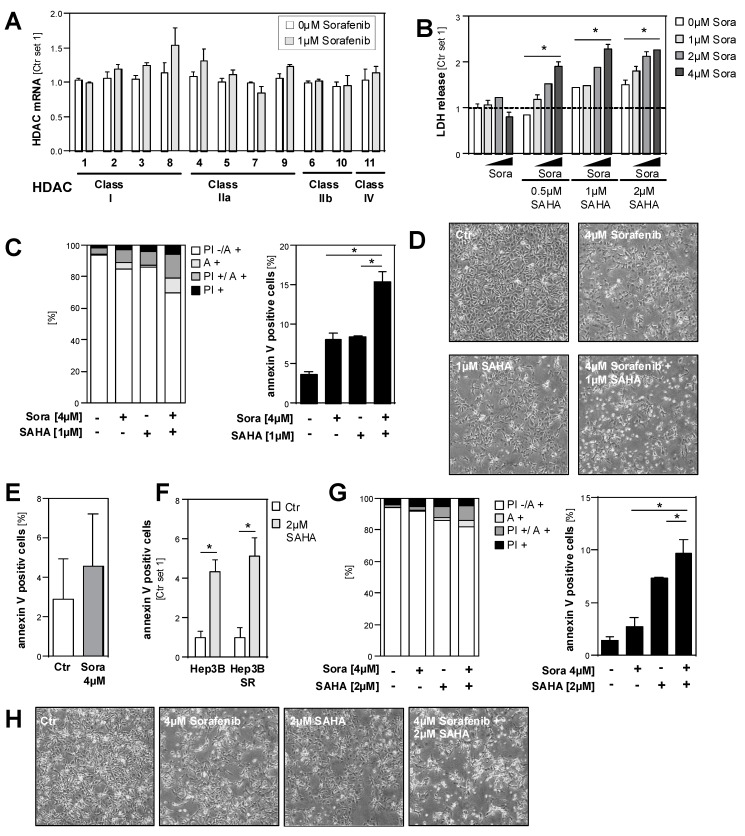
Effects of HDAC inhibition on HCC cells in combination with sorafenib. (**A**) The expression of different HDACs in HCC cells (Hep3B cells) after 24 h treatment with sorafenib (1 µM) or in control cells (Ctr.) treated with solvent (DMSO) only (set as 1). (**B**) Quantification of LDH release into the supernatants of HCC cells after 72 h treatment with sorafenib (Sora) and/or SAHA (Ctr. set 1). (**C**) Propidium iodide/annexin (PI/A) fluorescence-activated cell sorting (FACS) analysis of HCC cells after 72 h treatment with sorafenib (Sora) and/or SAHA (Ctr. set at a value of 1). Left panel: proportions of viable (PI -/ A -), early (A +) and late apoptotic (PI +/ A +) and necrotic cells (PI +); right panel: quantification of annexin-positive late apoptotic cells. (**D**) Representative microscopical images of HCC cells after 72 h treatment with sorafenib and/or SAHA. (**E**,**F**) Propidium iodide/annexin FACS analysis of (resistant) Hep3B cells and sorafenib-resistant Hep3B cells (Hep3B-SR) after 72 h treatment with sorafenib (Sora) (4µM; left panel) or SAHA (2 µM); quantification of annexin-positive cells. (Ctr. set 1). (**G**) Propidium iodide/annexin (PI/A) FACS analysis of Hep3B-SR cells after 72 h treatment with sorafenib (Sora) and/or SAHA. Left panel: proportions of viable (PI -/A -), early (A +) and late apoptotic (PI +/A +) and necrotic cells (PI +); right panel: quantification of annexin-positive late apoptotic cells. (**H**) Representative microscopical images of Hep3B-SR cells after 72 h treatment with sorafenib and/or SAHA. (*: *p* < 0.05).

Next, we wanted to analyze the effect of HDAC inhibition on HCC cells in combination with sorafenib, which is currently the only clinically established pharmacological therapy for HCC. Initially, we analyzed the effect of sorafenib on HDAC expression in HCC cells. For this analysis, we wanted to apply non-toxic sorafenib doses to avoid unspecific effects. In line with previous studies, the analysis of LDH release into the supernatant and microscopical analysis showed that sorafenib doses up to 1 µM did not affect the viability of HCC cells for up to 48h (Figure S4A). At this subtoxic dose, sorafenib did not significantly alter HDAC expression in HCC cells (Figure 7A). However, the combined treatment of HCC cells with 1 µM sorafenib and the HDAC inhibitor SAHA significantly enhanced LDH release into the supernatant as compared with treatment using the same SAHA or sorafenib doses alone (Figure 7B; Figure S4B). In combination with higher sorafenib doses (1 µM and 2 µM), the synergistic effect with SAHA was even more prominent (Figure 7B). FACS analysis confirmed that combined the treatment of HCC cells with sorafenib and SAHA synergistically induced cell death and apoptosis (Figure 7C,D; Figure S4C,D). These data indicate that HDACi can enhance the anti-tumorigenic efficacy of sorafenib. 

Next, we wanted to analyze whether HDACi can also affect efficacy for sorafenib treatment in sorafenib-resistant HCC cells. For this, we used sorafenib-resistant (SR) Hep3B cells (Hep3B-SR) that we had established in a previous study and that proliferate in the presence of up to 10 μM sorafenib [30]. FACS analysis revealed that a sorafenib dose of 4 µM did not significantly affect the number of annexin-positive Hep3B-SR (Figure 7E). SAHA had similar effects in Hep3B-SR as in non-resistant Hep3B cells (Figure 7F). However, the combination of SAHA and sorafenib treatments induced the number of annexin-positive cells significantly more than SAHA and sorafenib doses alone (Figure 5G). Microscopical analysis confirmed the increased combined toxic effect of SAHA and sorafenib on Hep3B-SR cells (Figure 7H). 

Together these data indicate that HDAC inhibition could be a potential therapeutic strategy to enhance the anti-tumorigenic efficacy of sorafenib in HCC cells.

In the search for the underlying mechanisms by which HDACi enhance the efficacy of sorafenib in HCC cells, we assessed the effect of HDACi on Kirsten rat sarcoma (KRAS) expression because we recently showed that wild type KRAS is dysregulated in HCC and promotes sorafenib resistance [30]. However, KRAS mRNA expression was not significantly altered in HDACi treated HCC cells (Figure S5). 

**Figure 8:**
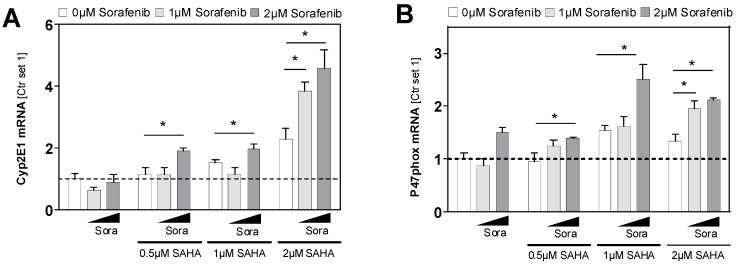
Cyp2E1 and p47phox expression 24 h after combined sorafenib and SAHA treatment. (**A**) Cyp2E1 and (**B**) p47phox mRNA expression in HCC cells treated with sorafenib (Sora) and/or SAHA. Control cells (Ctr.) treated with solvent (DMSO) only were set at a value of 1. (*: *p* < 0.05).

The sensitivity of human HCC cells to sorafenib has also been found to be associated with reactive oxygen species production and oxidative stress [31]. Cytochrome P450 2E1 (CYP2E1) is a critical mediator of oxidative stress in hepatocytes and HCC cells [32,33,34], and it has been shown that histone modification is involved in CYP2E1 gene expression in HCC cells [35]. Here, we found a dose-dependent induction of CYP2E1 expression by SAHA in HCC cells (Figure 8A). In contrast, a sorafenib dose of 2 µM had no significant effects on CYP2E1 expression levels (Figure 8A). However, combined treatment with sorafenib and SAHA had a significantly higher inducing effect on CYP2E1 expression than treatment with the same doses of sorafenib and SAHA alone (Figure 8A). Furthermore, the expression of p47phox, an established marker for oxidative stress [36], was significantly enhanced in HCC cells by combined treatment with sorafenib and SAHA compared to the effect of the two drugs alone (Figure 8B). Potentially, the inducing effects on CYP2E1 and oxidative stress, respectively, contribute to the impact of HDACi on the sensitivity of HCC cells to sorafenib.

## 3. Discussion

The first aim of this study was to systematically assess the expression levels of all classical HDACs in HCC cells and tissues compared to hepatocytes and non-tumorous liver tissues, respectively. We observed a significant upregulation of all members of the HDAC classes I, II and IV in HCC cell lines. The highest upregulation was observed for HDAC 2 (class I) as well as HDAC 7 and HDAC 9 (class II), with mRNA levels in part 100-fold higher than in hepatocytes. Furthermore, in three different HCC patient datasets, comprising a total of 705 patients, the expression of some HDACs was significantly enhanced in HCC compared with non-tumorous liver tissue. However, with the exception of HDAC 4 and HDAC 5, there was no unified picture of upregulation of the different HDACs in HCC compared to non-tumorous tissues in the three datasets. As one potential mechanisms, we observed a significant correlation of several HDACs with collagen expression in the non-tumorous liver tissues of HCC patients. This finding is in line with previous observations of pathologically altered histone acetylation and deacetylation regulated by HDACs in liver fibrosis [16]. Since HCC develops in most cases in a fibrotic liver, already increased HDAC levels in non-tumorous (fibrotic) liver tissue might explain that there was no unified picture of upregulation of HDACs in the different HCC patient datasets. Furthermore, it has to be noted that we did not observe a significant upregulation of HDACs in mouse models of early or pre-cancerous HCC stages. This indicates that the upregulation of HDAC expression occurs in most cases in advanced HCC stages. 

In addition to the non-tumorous liver tissues, in HCC tissues, we also observed a strong variation in the expression of all HDACs within different patients. Currently, we can only speculate whether factors such as the etiology of liver disease or other molecular mechanisms are causing these differences. One limitation of our study is that we focused our systemic analysis of the expression of the 11 classical HDAC members in HCC on the mRNA level. Still, previous studies assessing single HDACs have shown an upregulation of the protein expression of different HDACs, such as HDAC1 [6], HDAC2 [7], HDAC3 [37], HDAC4 [10], HDAC5 [8], HDAC9 [38], HDAC6 [39] and HDAC11 [40] in human HCC tissues compared with non-tumorous liver tissues. Furthermore, the analysis of the protein atlas (https://www.proteinatlas.org/, 09/2019) showed the strong expression of HDAC 1, 2 and 3 (class I), as well as HDAC 4 and 5 (class IIa) and HDAC 10 (class IIb), in most HCC tissues (Figure S6; for HDAC 7 and HDAC 11 no data were available in the protein atlas). Together, these findings confirm the upregulation of different HDACs in HCC also on the protein level.

Interestingly, we observed a significant correlation of the expression levels of all class IIa (HDAC 4, 5, 7, 9) as well as HDAC 10 and HDAC 11 levels in individual patients. This might indicate that there are high and low HDAC expressers and potentially patients in whom HDACs particularly affect disease progression or who particularly respond to therapy, respectively. However, these findings need to be confirmed in further studies with larger patient cohorts and it also has to be noted that HDACs and their activity are not only regulated at the transcriptional level [41]. Despite the concordance in the upregulation of most HDACs in HCC, it further has to be noted that it is likely that individual HDACs affect HCC progression differently. Here, we observed that in three large datasets of HCC patients, a high expression of HDAC class II members HDAC 4, HDAC 7 and HDAC 9 as well as HDAC class I members HDAC 1 and HDAC 2 significantly correlated with poor patient survival. Similarly, previous studies suggested high HDAC 1 [6,42] and HDAC 2 [6] expression as biomarkers for poor prognosis of HCC patients. A most recent study by Wang et al. based on immunohistochemical analysis found higher HDAC 4 expression in advanced HCC in 49 tissue samples examined [43]. Another recent study performed the HDAC 9 immunohistochemistry of 37 HCC tissue samples and found that patients with higher HDAC 9 expression had poorer prognosis [38]. 

In summary, these data indicate the high expression of some class I and class IIa HDAC members as predictors of poor outcome in HCC patients.

The second aim of this study was to analyze the effect of two different pharmacological HDAC inhibitors (HDACi) on HCC cell lines in functional in vitro analyses. Most previous studies assessing HDACi in HCC focused on (different modes of) cell killing. Here, we wanted to assess the functional effects of HDACi also in the subtoxic range to determine further insights on the role of HDACs in HCC. HDACi, TSA and SAHA, both significantly reduced proliferation, colony formation and migratory activity of HCC cells in subtoxic concentrations. Still, the effects of the two HDACi quantitatively differed, which may be related to their dissimilar effectiveness against different HDAC classes [13,14]. Moreover, we observed that SAHA but not TSA promoted features of cellular senescence in HCC cells. In some tumors such as rhabdomyosarcoma [44] and urothelial carcinoma [45], HDAC inhibition has been shown to affect senescence. Fan. et al. described that the knockdown of HDAC5 led to an up-regulation of p21 in HCC [8]. However, to the best of our knowledge, no further studies have assessed the impact of individual HDACs on cellular senescence and the effects of systemic HDACi on senescence has not yet been demonstrated in HCC cells. As comprehensively reviewed by Ramakrishna et al., senescence appears to act as a double-edged sword in HCC development and progression [46]. Further studies are necessary to elucidate whether this is also the case for SAHA effects on cellular senescence in HCC cells.

Finally, we analyzed the combined effect of HDACi and sorafenib on HCC cells. Previous studies have shown that the combination of sorafenib and HDACi resminostat improved overall survival compared to sorafenib monotherapy [47]. Moreover, another study found that the combination of sorafenib and resminostat helped to overcome sorafenib resistance [48]. Still, the molecular mechanism of these combined treatments is only incompletely understood. Yuan et al. proposed that HDACi could sensitize cancer cells to sorafenib treatment by regulating the acetylation level of Beclin-1 and herewith enhancing apoptosis [49]. He et al. showed that the combination of HDACi and sorafenib prevented HCC cell proliferation via implication in G0/G1 cell cycle arrest by the upregulation of the expression of p21 as well as the downregulation of certain cyclin-dependent kinases (CDK) and cyclins [50]. Lachenmayer et al. identified autophagy as a potential mechanism to make HCC cells susceptible to sorafenib treatment after resistance [51]. Soukupova et al. observed that resminostat shifted cancer cells to a more epithelial phenotype which might result in sensitization to sorafenib resistance [52]. Here, we observed that HDACi significantly enhanced the sensitivity of HCC cells to cell death induction by sorafenib. Moreover, we assessed HDACi effects in sorafenib-resistant HCC cells [30] and found that HDACi can overcome sorafenib resistance.

## 4. Materials and Methods 

### 4.1. Cells and Cell Culture 

The HCC cell lines HepG2 (ATCC HB-8065), PLC (ATCC CRL-8024), Hep3B (ATCC HB-8064) and Huh7 were cultured as described [53]. Primary human hepatocytes were isolated by the Biobank of the Department of General, Visceral and Transplant Surgery in Ludwig-Maximilians University using a two-step collagenase perfusion technique with modifications [54]. 

The murine Hepa129 cell line originates from a C3H/HeN mouse and was obtained from the NCI-Frederick Cancer Research and Development Center (DCT Tumor Repository). The murine Hepa1-6 cell line (ATCC CRL-1830) was also used. Isolation and culture of primary murine hepatocytes (PMH) were performed as described [55]. 

For stimulation experiments, cells were treated with trichostatin A (TSA) (Cayman Chemical, Ann Arbor, MI, USA), suberoylanilide hydroxamic acid (SAHA) (Cayman Chemical) and sorafenib (Biovision, Milpitas, CA, USA) at the concentrations and for duration as indicated.

### 4.2. Human Liver Tissues

Paired human HCC tissues and corresponding non-tumorous liver tissues were obtained from patients after partial hepatectomy. Double-coded human liver tissue used in this study was provided by the same Biobank as above. This Biobank operates under the administration of the Human Tissue and Cell Research (HTCR) Foundation. The framework of the HTCR Foundation [56], which includes obtaining written informed consent from all donors, has been approved by the ethics commission of the Faculty of Medicine at the LMU (approval number 025–12) as well as the Bavarian State Medical Association (approval number 11142) in Germany. 

### 4.3. In Silico Analysis

The "SurvExpress-Biomarker validation for cancer gene expression" database (http://bioinformatica.mty.itesm.mx:8080/Biomatec/SurvivaX.jsp) was used for the analysis of hepatocellular carcinoma LIHC-TCGA HCC, TCGA Liver Cancer and Hoshida Golub Liver GSE10143 datasets as described [29]. Kaplan Meier curves describe the overall survival of cancer patients with high HDACs expression as compared to low HDACs expression.

Oncomine^TM^ cancer microarray database analysis for HDAC gene expressions was performed using the website (https://www.oncomine.org/). The following datasets were used for the analysis: Roessler Liver2 [21]), Wurmbach Liver [22]) and Guichard Liver [23].

The University of California Santa Cruz (UCSC) Xena platform (available from: https://xenabrowser.net/) was used to calculate the correlation between HDAC and collagen I expression in non-tumorous liver tissue using a dataset from The Cancer Genome Atlas (TCGA) (n = 50). 

Furthermore, GEO datasets (GEO profiles) of (pre-)cancerous mouse models were used to analyses RNA expression levels for HDACs. Once, the murine Mdr2 knockout HCC model in both heterozygous (hetero, n = 6) and homozygous (homo, n = 6) knockouts was used. The Mdr2- KO mouse serves as a model for beta-catenin-negative subgroup of human HCCs characterized by down-regulation of multiple tumor-suppressor genes [27]. Moreover, the Trim24-KO murine HCC model was used to determine gene expressions in another GEO dataset in wild-type as compared to Trim24 knockout mice. Trim24 knockout mice spontaneously develop HCCs [28].

Immunohistochemical protein expression levels from HDACs in HCC tissue were depicted from the Protein Atlas Consortium website (https://www.proteinatlas.org/).

### 4.4. Analysis of mRNA Expression by Quantitative RT-PCR 

RNA isolation from cells and tissues and subsequent reverse transcription were performed as described [57]. Quantitative real-time-PCR was performed by applying LightCycler technology (Roche) as described [57], using specific sets of primers as listed in Table 3. Furthermore, for detection of human p47phox and murine HDAC9 QuantiTect Primer Assays (Quiagen, Hilden, Germany) were used. Amplification of cDNA derived from 18S rRNA was used for normalization.

### 4.5. Analysis of Cell Toxicity 

Cytotoxic effects were monitored by the analysis of lactate dehydrogenase (LDH) release into the supernatant with a colorimetry-based Cytotoxicity Detection Kit (Roche Applied Sciences, Indianapolis, IN, USA) following the manufacturer’s instructions. Further, cytotoxic effects were monitored by microscopical analysis. 

### 4.6. Quantification of Apoptosis

For apoptosis analysis, fluorescence-activated cell sorting (FACS) was performed using a conventional Annexin V-FITC Detection Kit (Promokine, Heidelberg, Germany). Annexin V and propidium iodide staining for the identification of viable, apoptotic and necrotic cells was conducted according to the manufacturer’s instructions and as described previously [30].

### 4.7. Clonogenic Assay

Clonogenic assays were performed to analyze stem cell behavior and attachment-dependent colony formation and the growth of cancer cells as described previously [30]. Briefly, cells were seeded at low density (1000 cells/well in a 6-well plate) in triplicates, treated with HDACi at the indicated concentrations and incubated for 10 days at 37 °C. Subsequently, cells were rinsed 2 times with PBS and fixed for 30 min with 6% v/v glutaraldehyde and stained with 0.5% crystal violet simultaneously, washed with tap water and dried before microscopical analysis. The number and size of colonies were calculated with CellSens Dimension Software (Olympus Soft Imaging Solutions GmbH, Münster, Germany).

### 4.8. Analysis of Cell Proliferation 

Cell proliferation was measured using the xCELLigence system (Roche) according to the manufacturer’s instructions. CyQUANT® NF Cell Proliferation Assay Kit was also used for proliferation analysis following the manufacturer’s instructions (Invitrogen, Carlsbad, CA, USA). Cells were seeded at a density of 1000 or 2000 cells/well in a 96-well plate.

### 4.9. Analysis of Cell Migration 

The migratory activity of HCC cells was quantified using the Cultrex 96 Well Cell Migration assay after treatment with HDAC inhibitors for 4 h (Trevigen, Gaithersburg, MD, USA) as described [58].

### 4.10. Analysis of Cellular Senescence

Cells were stained with Senescence β-Galactosidase Staining Kit (Cell Signaling, Danvers, MA, USA) following the manufacturer’s instructions. In brief, after 72 h treatment, cells were washed twice with PBS, fixed with the supplied fixation solution, washed twice again and incubated for 16 h with staining solution at 37 °C, washed again with PBS and water and subsequently dried overnight. 

### 4.11. Statistical Analysis

Values are presented as the mean ± SEM. A comparison between groups was made using the Student’s unpaired t-test or one-way ANOVA respectively. A *p* value < 0.05 was considered statistically significant. Correlation analysis was performed using univariate Pearson’s correlation coefficient (2-sided). A *p* value < 0.05 was considered statistically significant. All calculations were performed using the statistical computer package GraphPad Prism version 6.00 for Windows (GraphPad Software, San Diego, CA, USA).

## 5. Conclusions

Our study further confirms the potential beneficial use of HDACi in the treatment of HCC—alone or in combination with sorafenib. Importantly, in addition to promoting cell killing, HDACi appear to impair different facets of the tumorigenicity of HCC cells even at subtoxic doses. Generally, HDAC expression is increased in HCC but there appear to be differences between individual patients and HDAC classes. Furthermore, HDACi showed qualitatively and quantitatively different inhibitory effects on HCC cells in vitro. Together, the reason for these differences as well as their impact on HCC development and progression need to be further elucidated in future studies and could be exploited to develop more targeted therapeutic approaches.

## Figures and Tables

**Table 1:** Correlation of HDAC mRNA vs. collagen I (alpha 1) (Col I) in non-tumorous human liver tissues.

HDAC Class	Class I				Class IIa				Class IIb		Class IV
**HDAC**	**1**	**2**	**3**	**8**	**4**	**5**	**7**	**9**	**6**	**10**	**11**
**Col I**	r = 0.33 *p* = 0.04	r = 0.26 *p* = 0.07	r = 0.13 *p* = 0.75	r = 0.21 *p* = 0.14	r = 0.37 *p* = 0.01	r = 0.22 *p* = 0.41	r = 0.63 *p* < 0.01	r = 0.55 *p* = 0.01	r = 0.35 *p* = 0.03	r = −0.08 *p* = 0.08	r = 0.45 *p* < 0.01

Correlation analyses were conducted with the University of California Santa Cruz (UCSC) Xena platform using a dataset of 50 normal liver tissue samples to analyze the correlation between the RNA expression levels of all HDACs and collagen I. (r: Pearson correlation coefficient; blue boxes indicate correlations that are statistically significant (*p* < 0.05)).

**Table 2:** Correlation of the expression levels of different HDACs in human HCC tissues (n = 11).

HDAC Class		Class I				Class IIa				Class IIb		Class IV
	**HDAC**	**1**	**2**	**3**	**8**	**4**	**5**	**7**	**9**	**6**	**10**	**11**
**Class I**	**1**		0.41	0.60	0.29	0.56	0.42	0.62	0.41	0.55	0.56	0.79
	**2**			0.07	0.96	0.98	0.99	0.63	0.94	−0.12	0.93	0.80
	**3**				−0.14	0.15	0.07	−0.17	−0.11	0.59	0.28	0.32
	**8**					0.94	0.96	0.69	0.96	−0.18	0.86	0.74
**Class IIa**	**4**						0.97	0.72	0.95	0.01	0.96	0.90
	**5**							0.62	0.96	−0.14	0.92	0.80
	**7**								0.76	0.01	0.63	0.76
	**9**									−0.12	0.89	0.81
**Class IIb**	**6**										0.12	0.20
	**10**											0.87
**Class IV**	**11**											

The Pearson correlation coefficients are listed in the boxes. The blue boxes indicate correlations that are statistically significant (*p* < 0.05).

**Table 3:** Primer sequences for quantitative RT-PCR.

Gene	Forward (5’-3’)	Reverse (5’-3’)
**human primer**		
***Cyp2E1***	CCCTGCAACGTCATAGCC	TTTCCACGAGCAGGCAGT
***HDAC1***	ACTACGACGGGGATGTTGGA	CAGCATTGGCTTTGTGAGGG
***HDAC2***	GCTCTCAACTGGCGGTTCAG	AGCCCAATTAACAGCCATATCAG
***HDAC3***	GGCCTATTTCTACGACCCCG	TGGTATGGCTTGAAGACGATCA
***HDAC8***	TGTGACTCCCTGGCCAAGAT	AGATGCTTCATCTCTCATCTGCT
***HDAC4***	CCACCTCACTCCCTACCTGA	CCCAGGCCTGTGACGAG
***HDAC5***	GCACCATCGCTGAGAATGGC	GGGAGGCAGTGAGGTGTGAG
***HDAC7***	AGCAGCTTTTTGCCTCCTGTT	TCTTGCGCAGAGGGAAGTG
***HDAC9***	AGGCTCTCCTGCAGCATTTATT	AAGGGAACTCCACCAGCTACAA
***HDAC6***	CTGGCGGAGTGGAAGAACC	GGGGAACGGCTCCCTTTTTA
***HDAC10***	TGGCCTTTGAGTTTGACCCTG	GGCTGAGTCAAATCCTGCCG
***HDAC11***	CCCCTTGGTCATGGGATTT	CATCCACACCAGTGCCTATAGC
***KRAS***	AACAGGCTCAGGACTTAGCAA	TCATCAACACCCTGTCTTGTCT
***p21***	CGAGGCACCGAGGCACTCAGAGG	CCTGCCTCCTCCCAACTCATCCC
***PML***	CAGCAGTGAGTCCAGTGACC	GACACGGCCTTGGAGTAGAT
**murine primers**		
***HDAC1***	ACTACGACGGGGATGTTGGA	CAGCATTGGCTTTGTGAGGG
***HDAC2***	AGGTGAAGGAGGTCGTAGGAA	TCTGACTTGGCTCCTTTGGG
***HDAC3***	ATGCCTTCAACGTGGGTGAT	CAGAAGCCAGAGGCCTCAAA
***HDAC8***	GCAATGAGCCCCACCGAATC	TCCACAAACCGCTTGCATCA
***HDAC4***	GGGGAGCAGCATCATGGTTCA	GGTCCCAGCTGCGTAAACT
***HDAC5***	GGTCGTAAAGCCACACTGGA	TGTCACTGTCCACCCCAATG
***HDAC7***	TAGCCAGCAGTGTGGTCAAG	GGGCAGGCTGTAGGGAATAC
***HDAC6***	CACCGCATTCAGAGGGTTCT	CCTTAAGGTGGGGCCAGAAG
***HDAC10***	CTCCACGCACTGTCTAAGCA	GTGAAAGGTGTCCGGGTGAA
***HDAC11***	CTGGCCCATCGTGTACTCAC	GTTGAGATAGCGCCTCGTGT
***18S***	AAACGGCTACCACATCCAAG	CCTCCAATGGATCCTCGTTA

## Supplementary Materials

The following are available online at https://www.mdpi.com/2072-6694/11/10/1587/s1, Figure S1: HDAC expression in tumorous liver tissue samples of HCC patients, Figure S2: HDAC expression levels and prognosis of HCC patients, Figure S3: Effects of HDAC inhibition on the viability of HCC cells, Figure S4: Effects of sorafenib alone and in combination with SAHA on the viability of HCC cells, Figure S5: Kras expression in HCC cells after SAHA and TSA treatment, Figure S6: Immunohistochemical staining of HDACs in HCC tissues from Human Protein Atlas.
