# Supplementary material for: Histone Deacetylase Expressions in Hepatocellular Carcinoma and Functional Effects of Histone Deacetylase Inhibitors on Liver Cancer Cells In Vitro
Source: Cancers (Basel). 2019 Oct 18;11(10):1587. doi: 10.3390/cancers11101587 (PMC6826839; doi:10.3390/cancers11101587)
Supplement: Supplementary file 1 [file cancers-11-01587-s001.pdf]

Supplementary Figures

SFigure 1

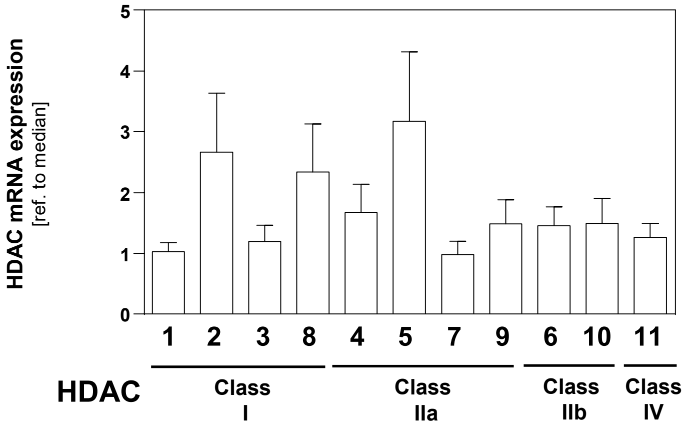

SFigure 1: HDAC expression in tumorous liver tissue samples of HCC patients.
HDAC mRNA expression in 11 human HCC tissue samples referred to the median.

SFigure 2

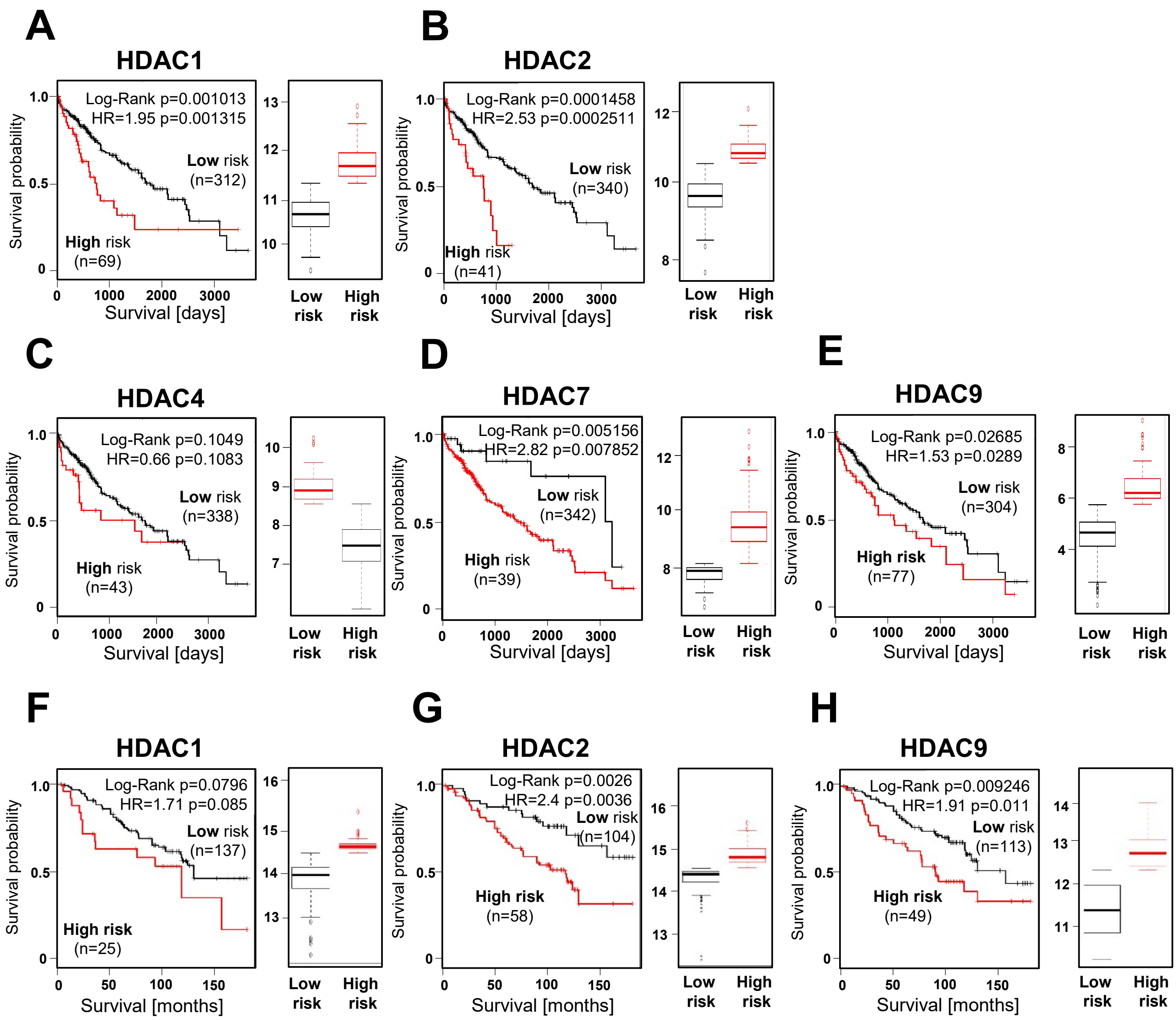

SFigure 2: HDAC expression levels and prognosis of HCC patients.
(A-H) Representative Kaplan-Meier survival curve analysis was performed using SurvExpress Biomarker validation database for a TCGA HCC (TCGA Liver cancer) dataset (n=422) (A-E) and Hoshida Golub Liver GSE10143 dataset (n=162) (F-H) for overall survival (left panel) with stratification into `low risk` and `high risk` group based on prognostic index for HDAC 1, 2, 4, 7, and 9, respectively and quantification of corresponding mRNA expression (right panel). No data for HDAC4 and HDAC7 were available in the Hoshida Golub Liver dataset.

SFigure 3

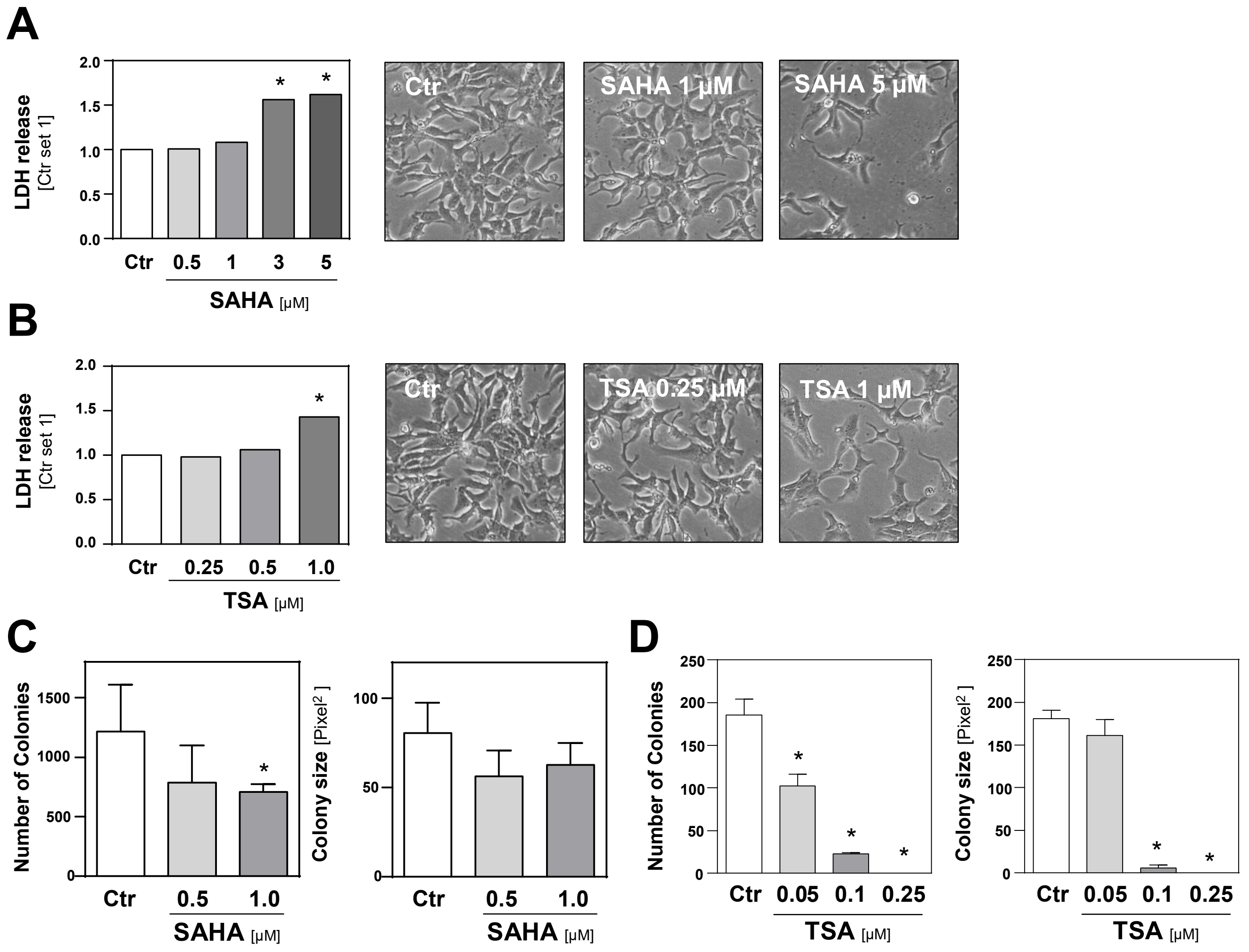

SFigure 3: Effects of HDAC inhibition on the viability of HCC cells.
(**A,B**) Lactate dehydrogenase (LDH) release into the supernatant (left panel) and microscopical images (right panels) of human HCC cells (eg. Hep3B) after 72h treatment with different doses of SAHA (A) or TSA (B) (Ctr. set 1). (**C,D**) Anchorage-dependent clonogenic assay with HepG2 cells after SAHA (C) and Hep3B after TSA (D) treatment. Quantification of colony numbers (left panel) and colony size (right panel). (*: $p < 0.05$ compared to Ctr.)

SFigure 4

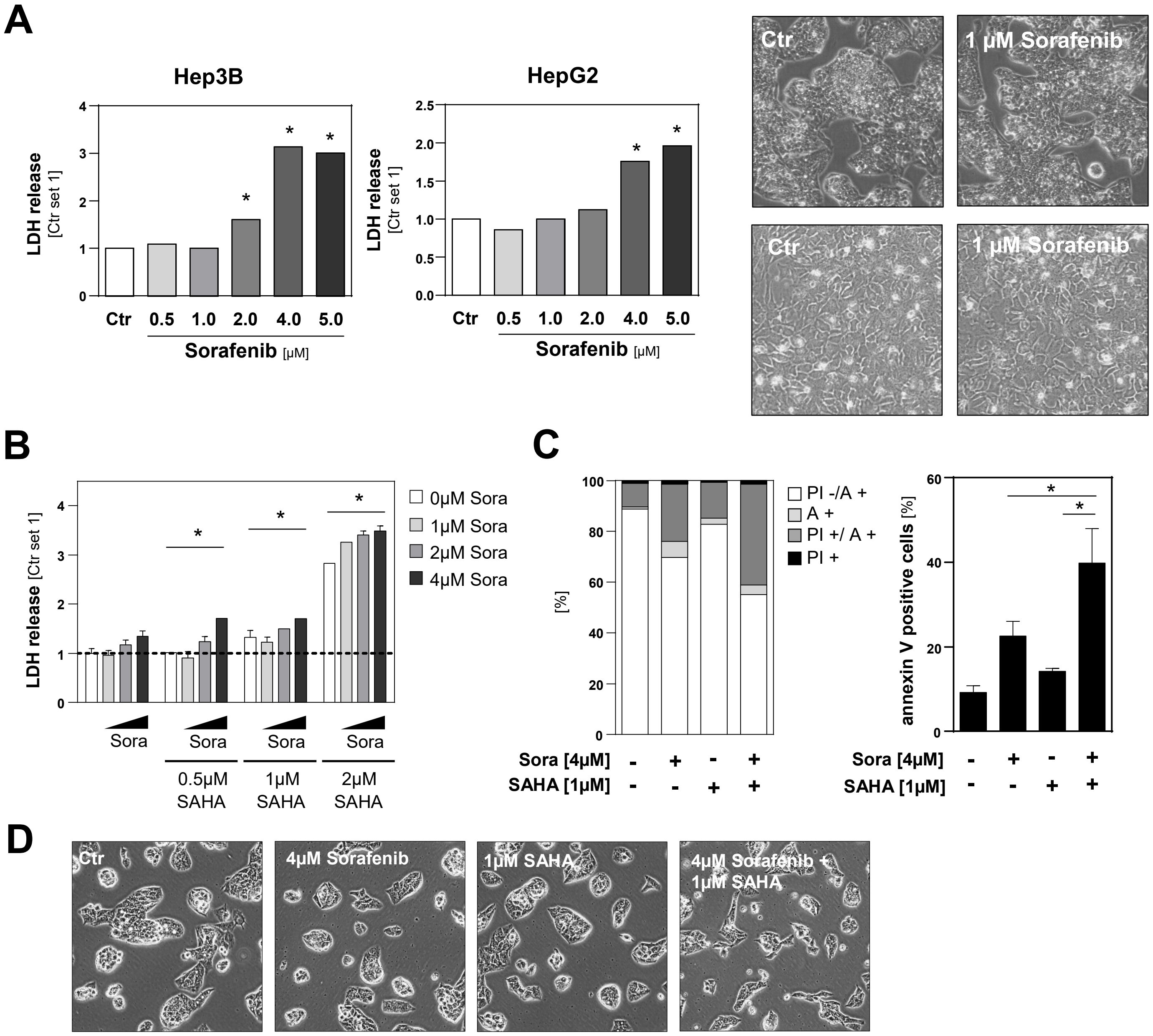

SFigure 4: Effects of sorafenib alone and in combination with SAHA on the viability of HCC cells.
(**A**) LDH release into the supernatant (left panels) and microscopical images (right panels) of human HCC cells (Hep3B, HepG2) after 48h treatment with increasing doses of sorafenib (Ctr. set 1). (**B**) Quantification of LDH release into the supernatants of HepG2 cells after 72h treatment with sorafenib (Sora) and/or SAHA (Ctr. set 1). (**C**) Propidium iodide/annexin (PI/A) FACS analysis of HepG2 cells after 48h treatment with sorafenib (Sora) and/or SAHA. Left panel: proportions of viable (PI -/ A -), early (A +) and late apoptotic (PI +/ A +) and necrotic cells (PI +); right panel: quantification of annexin-positive late apoptotic cells. (**D**) Representative microscopical images HCC cells after 72h treatment with sorafenib and/or SAHA. (*: $p < 0.05$ compared to Ctr.)

SFigure 5

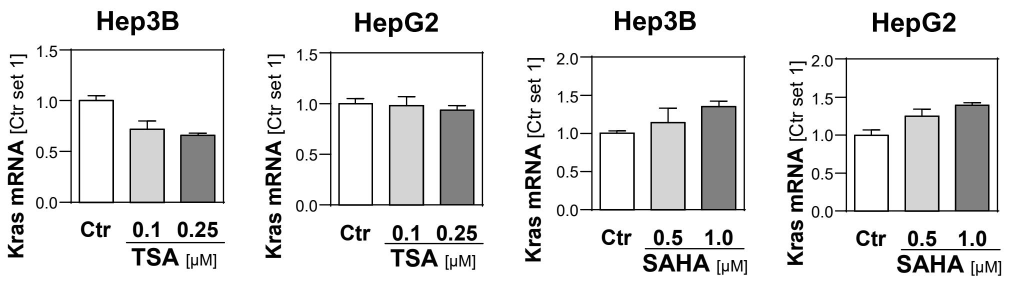

SFigure 5: Kras expression in HCC cells after SAHA and TSA treatment.
Kras mRNA expression in human HCC cells (Hep3B, HepG2) treated with SAHA or TSA for 24h with increasing doses. Control cells (Ctr.) treated with solvent (DMSO) only were set as 1. (*: p <0.05).

SFigure 6

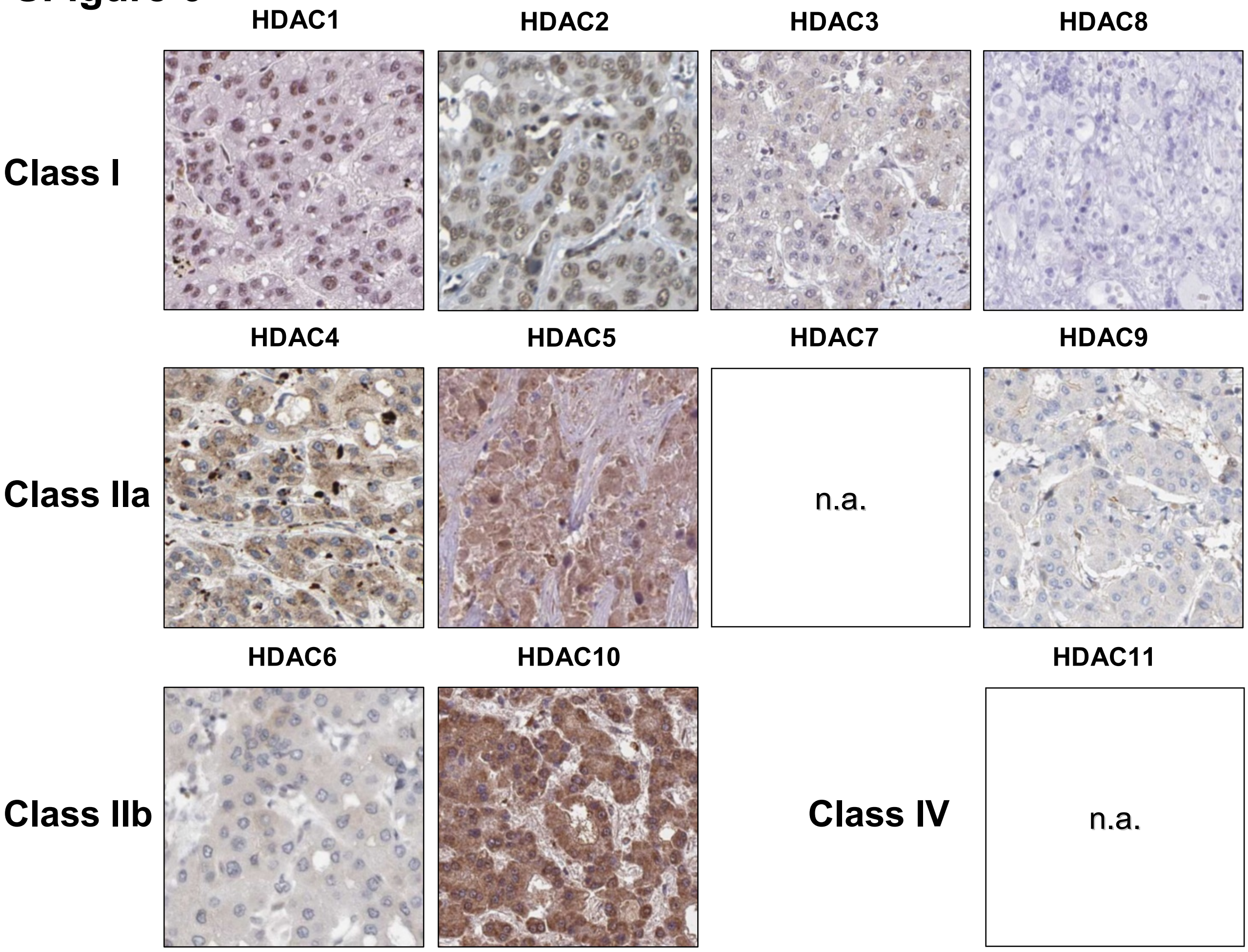

SFigure 6: Immunohistochemical staining of HDACs in HCC tissues from Human Protein Atlas.
HDAC immunohistochemistry available from Human Protein Atlas Consortium website for HCC tissue. No staining was available for HDAC7 and HDAC11. (https://www.proteinatlas.org/; September 2019)
